# Identification and characterization of lipoxygenase (LOX) genes involved in abiotic stresses in yellow horn

**DOI:** 10.1371/journal.pone.0292898

**Published:** 2023-10-13

**Authors:** Fang Hu, Yunxiang Zhang, Jinping Guo

**Affiliations:** 1 The College of Forestry, Shanxi Agricultural University, Jinzhong, Shanxi, China; 2 Shanxi Key Laboratory of Functional Oil Tree Cultivation and Research, Jinzhong, Shanxi, China; Nuclear Science and Technology Research Institute, ISLAMIC REPUBLIC OF IRAN

## Abstract

Lipoxygenase (LOX) gene plays an essential role in plant growth, development, and stress response. 15 LOX genes were identified, which were unevenly distributed on chromosomes and divided into three subclasses in this study. In promoter region analysis, many cis-elements were identified in growth and development, abiotic stress response, hormonal response, and light response. qRT-PCR showed that the LOX gene showed tissue specificity in seven tissues, especially *XsLOX1*, *3*, and *7* were relatively highly expressed in roots, stems, and axillary buds. The different expression patterns of LOX genes in response to abiotic stress and hormone treatment indicate that different *XsLOX* genes have different reactions to these stresses and play diversified roles. This study improves our understanding of the mechanism of LOX regulation in plant growth, development, and stress and lays a foundation for further analysis of biological functions.

## Introduction

Lipoxygenases (LOXs) are widespread in plants and animals [1], catalyzing the oxidation of polyunsaturated fatty acids (PUFA) in complex eukaryotes [2]. Based on sequence similarity, the 13-LOXs can be further partitioned into two divisions: Type I and Type II [3]. LOXs encompass two significant domains, one at the C-terminal lipoxygenase domain and another at the N-terminal polycystic protein-1, lipoxygenase, α-toxin (PLAT) domain [4].

With sequencing and functional genomics progress, studies on LOX-encoding genes are burgeoning in various plant species. Numerous previous studies have shown the function of LOXs in signaling transduction, development, and aging in plants [3, 5–8], particularly in the face of biological [7, 9–11] and environmental stresses [12–21]. Notably, *AtLOX1* and *AtLOX5* are associated with lateral root development [22]; *AtLOX2*, *AtLOX3*, *AtLOX4*, and *AtLOX6* are implicated in JA biosynthesis. Moreover, *AtLOX2* can be induced in response to damage. *AtLOX6* is involved in drought tolerance [17], while *AtLOX3* and *AtLOX4* have been implicated in flower development [23]. The peanut lipoxygenase *AhLOX29* [20] and oriental melon *CmLOX10* [24] play a role in drought tolerance, the pepper lipoxygenase *CaLOX1* [25] and the rice *OsLOX10* [26] affect and resistance to high salinity stress. The persimmon *DkLOX3* [18] and passiflora edulis *PeLOX4* [27] promoted ripening.

Yellow horn (*Xanthoceras sorbifolium* Bunge) is an oil-rich seed shrub with important economic value. Due to its strong adaptability to various abiotic stress conditions such as saline-alkali environments, deserts, and arid areas, it can help to eliminate desertification and erosion and be planted as an ornamental tree. However, the systematic identification and classification of the LOX genes in yellow horn have yet to be reported. They are following the accomplishment of genome-wide sequencing, which makes it possible to pinpoint and analyze the LOX family members in yellow horn. Fifteen LOX genes have thus been identified, and each has undergone an analysis of its phylogenetic relationships, chromosomal distribution, homology, gene structure, subcellular localization, conserved protein motifs, cis-acting elements, and expression patterns. These studies provide novel insights into the exploration of essential functional genes for regulating plant growth, development, and responding to stresses in yellow horn.

## Materials and methods

### Identification and classification

We downloaded *Xanthoceras sorbifolium* genome data from the GigaScience GigaDB repository. The protein sequences of LOX in *Arabidopsis thaliana*, *Populus trichocarp*, and *Oryza sativa* were retrieved from the Phytozome website (https://phytozome.jgi.doe.gov/). We submitted the protein sequences as queries in the BLAST search to identify members of the gene family. A Hidden Markov Model (HMM) profile (PF00305) was obtained from the Pfam database for gene family identification with HMMER3.0. An E-value cutoff was 0.001, and default parameters were taken. Afterward, the conserved domains of candidate genes were confirmed via the NCBI Database (https://www.NCBI.nlm.nih.gov/cdd) to predict the conserved functional domains. The computing tool PI/MW of ExPASy (https://web.expasy.org/compute_pi/) was utilized to determine the PI and MW of soybean LOXs. We used WoLF PSORT online tool to predict the subcellular localization of LOX genes.

### Phylogenetic analysis

The clustalW program produced alignments among various *XsLOX* candidates applying the default parameters [28]. Phylogenetic trees were built according to the maximum likelihood method using MEGA 7.0 [29]. Nodal support was assessed with 1,000 bootstrap replicates, and protein sequences were classified into their respective 9- and 13-LOX categories. Subsequently, the phylogenetic tree was visualized in iTOL.

### Gene structure and conserved motif

The conserved protein sequence motifs of the *XsLOXs* were analyzed using Multiple Em for Motif Elicitation web tool (https://meme-suite.org/meme/tools/meme) with the parameters maximum of 10 motifs and a width ranging from 6 and 50 amino acid residues [30]. The structures of genes were mapped through the online Gene Structure Display Server [31], while the phylogenetic tree and conserved motifs were integrated and visualized via Tbtools [32].

### Chromosome distribution and gene duplication

The physical location information of all *XsLOX* genes was determined from the yellow horn genomic database. We investigated collinearity relationships and gene duplication events through the MCScanX (Multiple Collinear Scanning Toolkits) [33] and displayed them with circus software of TBtools software.

Dual Synteny Plotter software was adopted to generate the syntenic relationships between *XsLOX* and LOX genes from *Arabidopsis thaliana*, *Populus trichocarpa*, and *Oryza sativa*; Ka and Ks substitutions, as well as the Ka/Ks ratio, were respectively determined using KaKs Calculator software 2.0 [34].

### Analysis of cis-acting regulatory elements in *XsLOX* genes

The 2.0 kb upstream promoter sequences of each *XsLOX* genes were uploaded to the online site PlantCARE (http://bioinformatics.psb.ugent.be/webtools/plantcare/html/) to identify the cis-acting regulatory elements.

### Plant materials and stress treatments

Yellow horn seedlings grew in a greenhouse at 25°C for a month. Healthy seedlings were treated at 4°C, 150 mM NaCl, and 150 mM Na_2_CO_3_ (pH 9.5) as cold, salt, and saline-alkali stress, respectively. The leaves were collected at low temperatures at 0 h, 4 h, 12 h, and 24 h, and salt, and saline-alkali stress at 0 h, 4 h, and 24 h for transcriptome sequencing. All transcriptome data with accession numbers (salt, at SRX7830132—SRX7830143; alkali and low-temperature stress, at SRX8911738—SRX8911752) were available from the NCBI SRA database. We mapped these data to the *X*.*sorbifolium* reference genome [35] and evaluated *XsLOX* gene expression levels using Cufflinks software [36]. A heat map was generated using TBtools.

Four-weeks-seedlings were treated with 100 mM NaCl, 100 μM ABA, 200 μM Salicylic Acid (SA), 10% Polyethylene Glycol 6000 (PEG6000), 100 μM Gibberellin A3 (GA3), and 4°C as cold stress respectively. Twenty-four hours after exposure to the abiotic and hormonally medicated treatments, leaves were collected. Various tissues were obtained from the experiment plot to procure specimens at the flowering stage from triennial plants. Before RNA extraction, the collected samples were frozen directly in liquid nitrogen and stored at -80°C.

qRT-PCR was performed with Fast SYBR Green PCR kit on an ABI 7,500 apparatus (Applied Biosystems, Carlsbad, CA, USA), and PCR primer pairs for candidate genes were designed by using Primer3 (**S1 Table**). UBC2 (EVM0006862) was used as an internal control. Finally, the comparative 2^−ΔΔCt^ method computed the relative expression, and each sample was replicated three times.

## Results

### For identification and classification of *XsLOX*

15 *XsLOX* gene members were identified from yellow horn genome through HMM-scan software and BLAST search at the genome-wide level. We named these LOX genes *XsLOX1* to *XsLOX15* according to their order on the chromosome positions. The fundamental properties of *XsLOX* genes were then analyzed and represented in **S2 Table**, including gene ID, gene name, position on the chromosome, length of coding sequence (CDS), length of the corresponding protein, molecular weight (MW), theoretical isoelectric point (pI), and the predicted subcellular localization. The related proteins of the *XsLOX* genes ranged in length from 831 (*XsLOX1*) to 927 (*XsLOX11*) amino acids, with their respective molecular weights and isoelectric points varying from 94.82 kDa to 104.52 kDa and from 5.33 (*XsLOX14*) kDa to 7.7 (*XsLOX11*) kDa, respectively. Most *XsLOX* genes were predicted to target the cytoplasm and chloroplast, while only one gene was localized within the nucleus.

### Phylogenetic analysis of *XsLOX*

**Fig 1:**
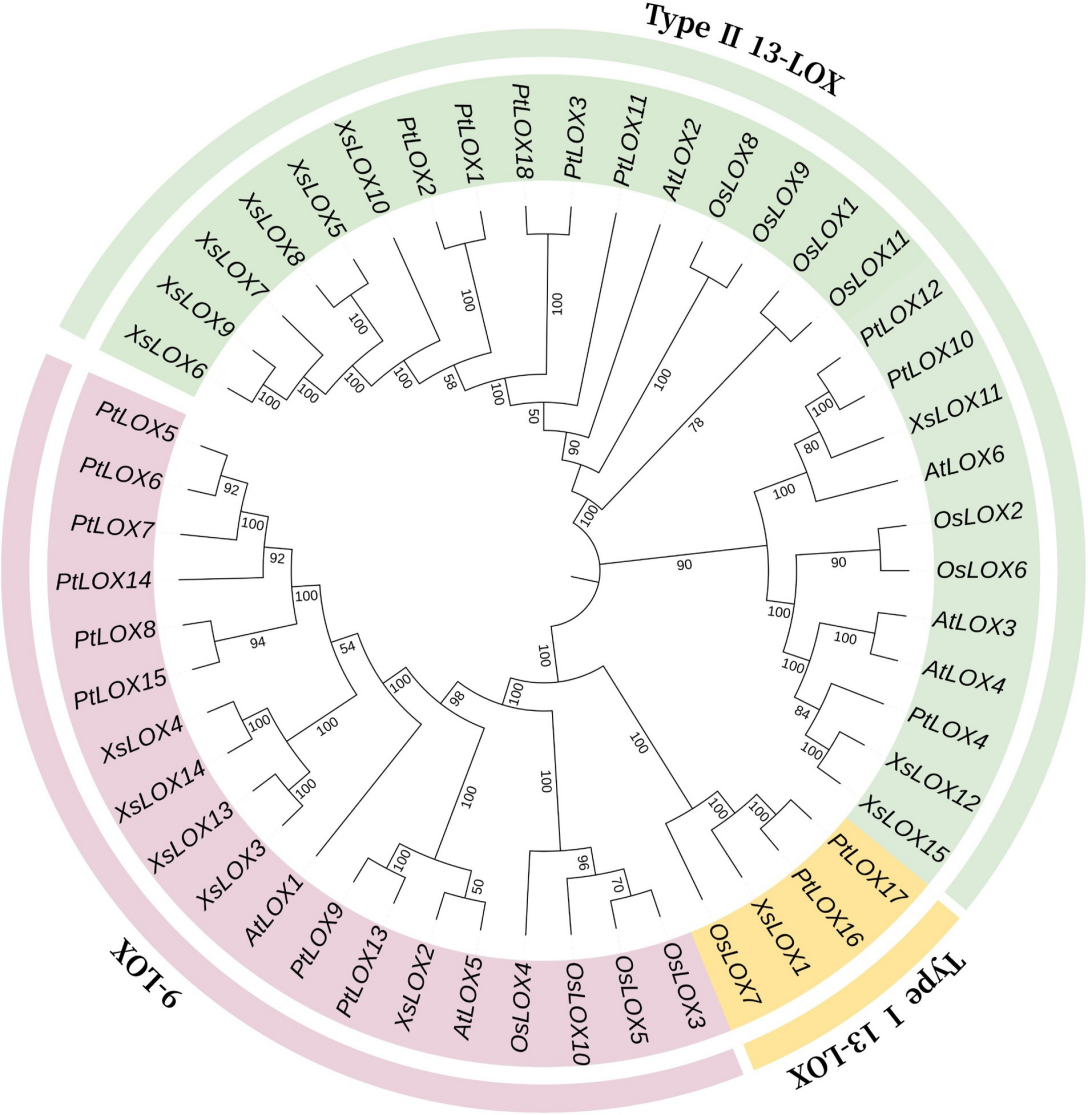
Phylogenetic tree of LOX genes with other plants. The phylogenetic tree in the figure also distinguished the LOX genes of the various plants with distinct colors (9-LOX: purple, type I 13-LOX: orange, and type II 13-LOX: green). *AtLOX*: *Arabidopsis thaliana*. *PtLOX*: *Populus trichocarpa*. *OsLOX*: *Oryza sativa*.

A neighbor-joining phylogenetic tree was constructed with 15 LOX sequences (from *X*.*sorbifolium*), 6 LOX sequences (from *A*. *thaliana*), 11 LOX sequences (from *O*. *sativa*), and 18 LOX sequences (from *P*. *trichocarpa*). Utilizing this tree, researchers were able to trace the evolutionary history of these genes (**Fig 1**). It showed that *XsLOX* genes were classified into three categories: type I 13-LOX, type II 13-LOX, and 9-LOX. Five and nine *XsLOXs* were assigned to the 9-LOX subfamily and type II 13-LOX subfamily, respectively. The type I 13-LOX subfamily was seen to be the minor branch occupied by a single gene.

### Analysis of gene structure and conserved motif of *XsLOX*

**Fig 2:**
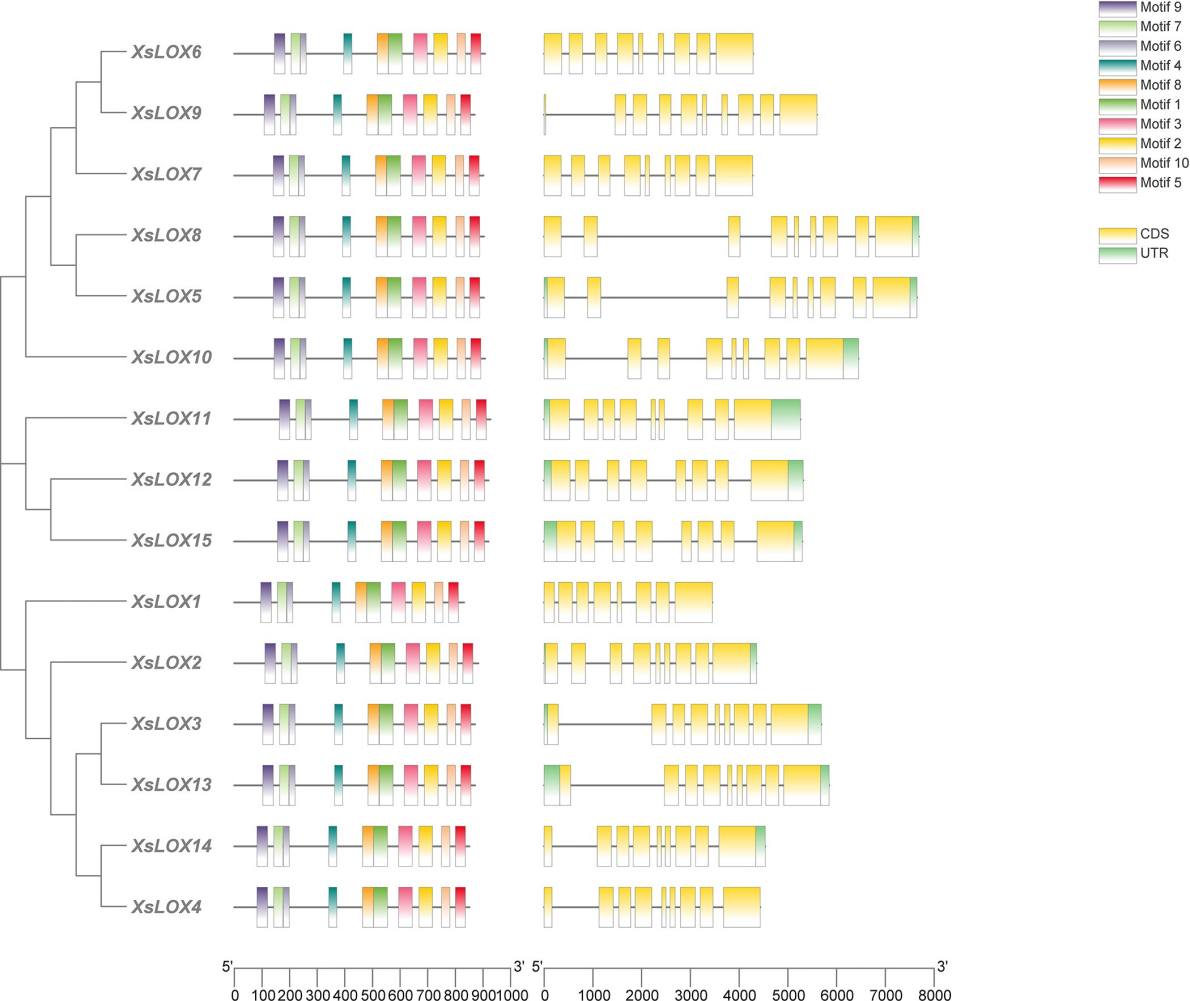
Phylogenetic relationships, conserved domain regions, gene structure, and motif patterns of LOX genes in yellow horn. Clustal W and MEGA 7 were employed to realign the complete amino acid sequence and formulate the phylogenetic tree. The untranslated region (UTR), exons, and introns were depicted as green boxes, yellow boxes, and grey lines, respectively.

We observed that the number of exons and introns varied among *XsLOX* genes and that conserved domains were broadly distributed (**Fig 2**). It was apparent that all *XsLOX* genes featured exons, nine being the most widespread within the family. *XsLOX9* was the most abundant in exonic components compared to the other genes. Using MEME, we also revealed that the subfamily included ten conserved motifs in the LOX proteins. All ten motifs were present in all *XsLOX* proteins, and they had the same organization, indicating a considerable level of sequence similarity and conservation within *XsLOX*. Motif 1, consisting of HIS-(X) 4 -HIS-(X) 4 -HIS-(X) 17 -HIS-(X) 8 -HIS, is purported to be an integral pattern influencing the activity and stability of the lipoxygenase (LOX) enzyme [5, 37] (**S3 Table**).

### Chromosome distribution and duplication of *XsLOX*

**Fig 3:**
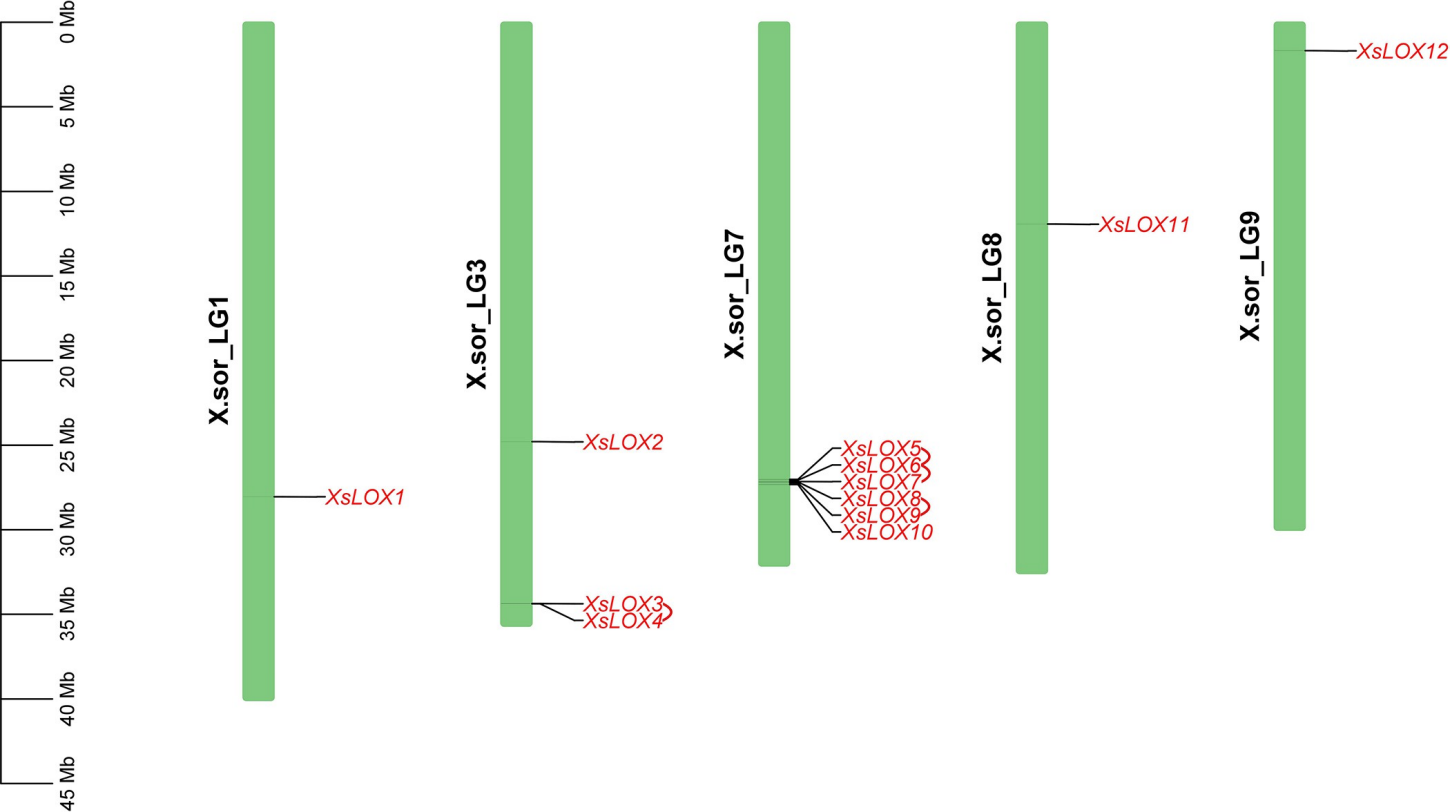
Further illustrates the alignment of *XsLOX* genes in 5 chromosomes, expressing the gene arrangements in relative proportions. Each line on the chromosome represents the fragment of a gene. The correlation between the two gene headers indicates that they are matched duplicates.

The map chart software was employed to specify the genes’ locations on chromatids. Subsequently, the *XsLOX* genes were categorically renamed according to their chromosomal distribution. Data analysis suggested substantial divergence in the figure of *XsLOX* genes across chromosomes (**Fig 3**). Chromosome number 6 contained the most *XsLOX* genes, whereas chromosomes 1, 8, and 9 each harbored only one gene. Interestingly, the research did not demonstrate a conspicuous association between the length of chromosomes and the proliferation of *XsLOX* genes. The aggregate elucidation from the investigation indicated an asymmetric diffusion of *XsLOX* genes on the chromosomes.

Seven *XsLOX* genes were observed in the MCScanX package to have experienced successive duplication events across chromosomes, with three pairs constituting the 13-LOX II branch and one belonging to the 9-LOX branch in yellow horn (**Fig 3**). This duplication is correlated with an increase in the *XsLOX* family, and Ka/Ks ratios were calculated to explore the effects of selection on this gene family following replication (**S4 Table**). These figures suggest that the genes within the LOX genes are evolving under negative selection pressure, as evidenced by the Ka/Ks ratios that range from 0.24 to 0.39.

**Fig 4:**
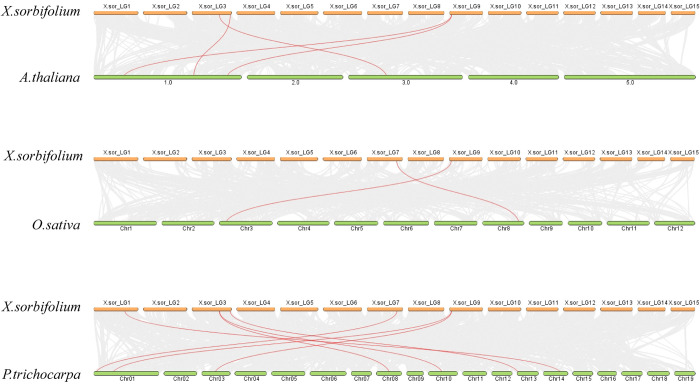
Homolinear LOX gene analysis between *X*.*sorbifolium*, rice, *A*. *thaliana*, and *P*. *trichocarpa*. A homology-based LOX gene analysis was performed among *X*.*sorbifolium*, rice, *A*. *thaliana*, and *P*. *trichocarpa*. The gray lines in the background indicated collinear blocks between *X*.*sorbifolium*, rice, A. thaliana, and *P*. *trichocarpa*, while the red line signified the collinear LOX gene pairs.

Every chromosome is denoted with a numerical label at the right. A syntenic map of three species (**Fig 4**) can be utilized to investigate the evolutionary relationship of the *XsLOX* genes. Collinearity analysis revealed that the four *XsLOX* genes were homologous with A. thaliana genes, two *XsLOX* genes with rice genes, and seven *XsLOX* genes with *P*. *trichocarpa* genes on the chromosome.

## Analysis of cis-acting regulatory elements (CREs) of *XsLOX*

**Fig 5:**
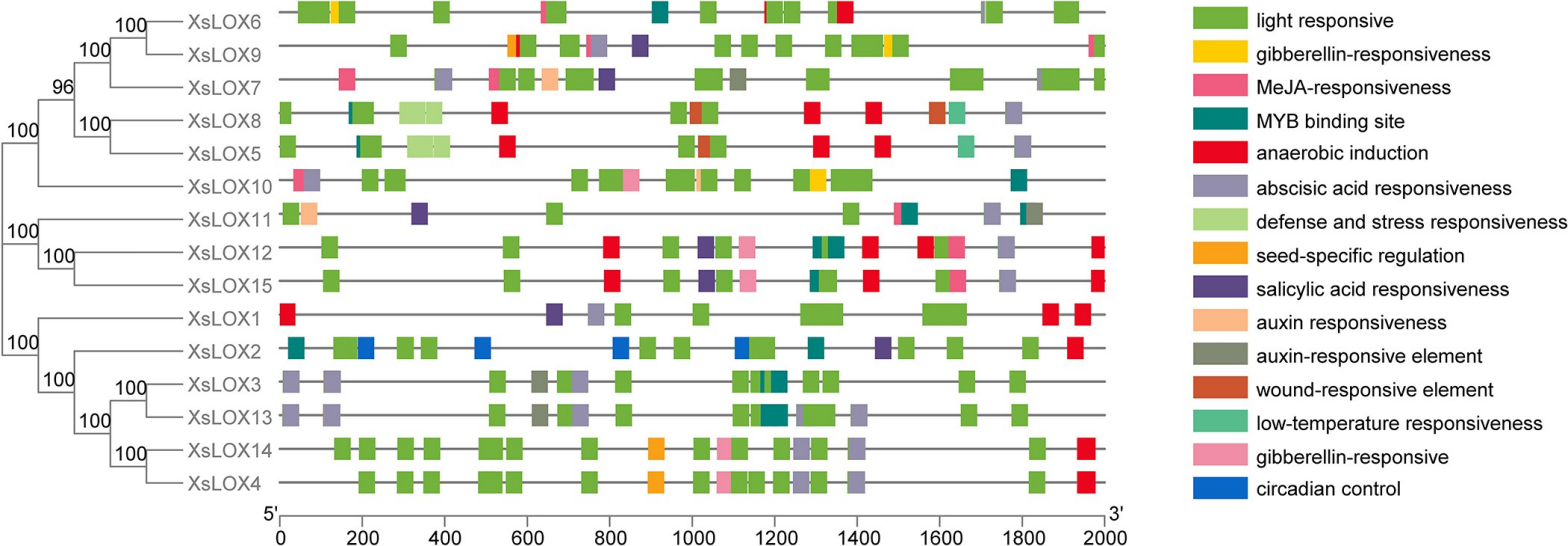
Phylogenetic tree and distribution of cis-acting elements in the *XsLOX* gene promoter regions. Different colors in the boxes represent the cis-acting elements.

Cis-regulatory elements comprise non-coding DNA sequences, which can influence regulatory networks, and are bound by transcription factors to regulate gene expression. CREs have an integral role in regulating gene expression due to embedding in non-coding DNA regions, which are predominantly bound by transcription factors to modulate the transcription of adjacent genes. To uncover the cis-acting elements of *XsLOX* genes, the PlantCare program was employed to detect the 2000-bp genomic DNA upstream sequence of the start codon. The analysis showed that the cis-acting elements in the promoter regions were mainly associated with MeJA, ABA, IAA, GA, and SA, indicating that *XsLOX* genes may be involved in plant hormone signaling pathways (**Fig 5**).

Moreover, the CREs of genes are linked to various conditions such as stress tolerance, healing, defense, hypothermia, anaerobic conditions, and hypoxia. Our examination of cis-regulatory elements uncovered discrepancies in the number of CREs among the five gene families: 119 primarily had abscisic acid response elements, 101 possessed MeJA response elements, 93 contained gibberellin response elements, 37 had low-temperature responsive elements, 145 had light-responsive features. Interestingly, soloist genes had fewer CREs when compared to other genes that were associated with hormone pathways and stress responses. Analysis of cis-regulatory factors revealed differences in the number of CREs among the five gene families. The outcome implies that *XsLOX* is essential for the growth and assimilation of plants in diverse environmental conditions.

### For identification and classification of *XsLOX*

#### Transcriptome analysis of LOX

To comprehend the expression pattern of XsLOX genes, we analyzed RNA-seq data under salt, alkali, cold, and drought stress. The heat map was constructed with TBtools software.

**Fig 6:**
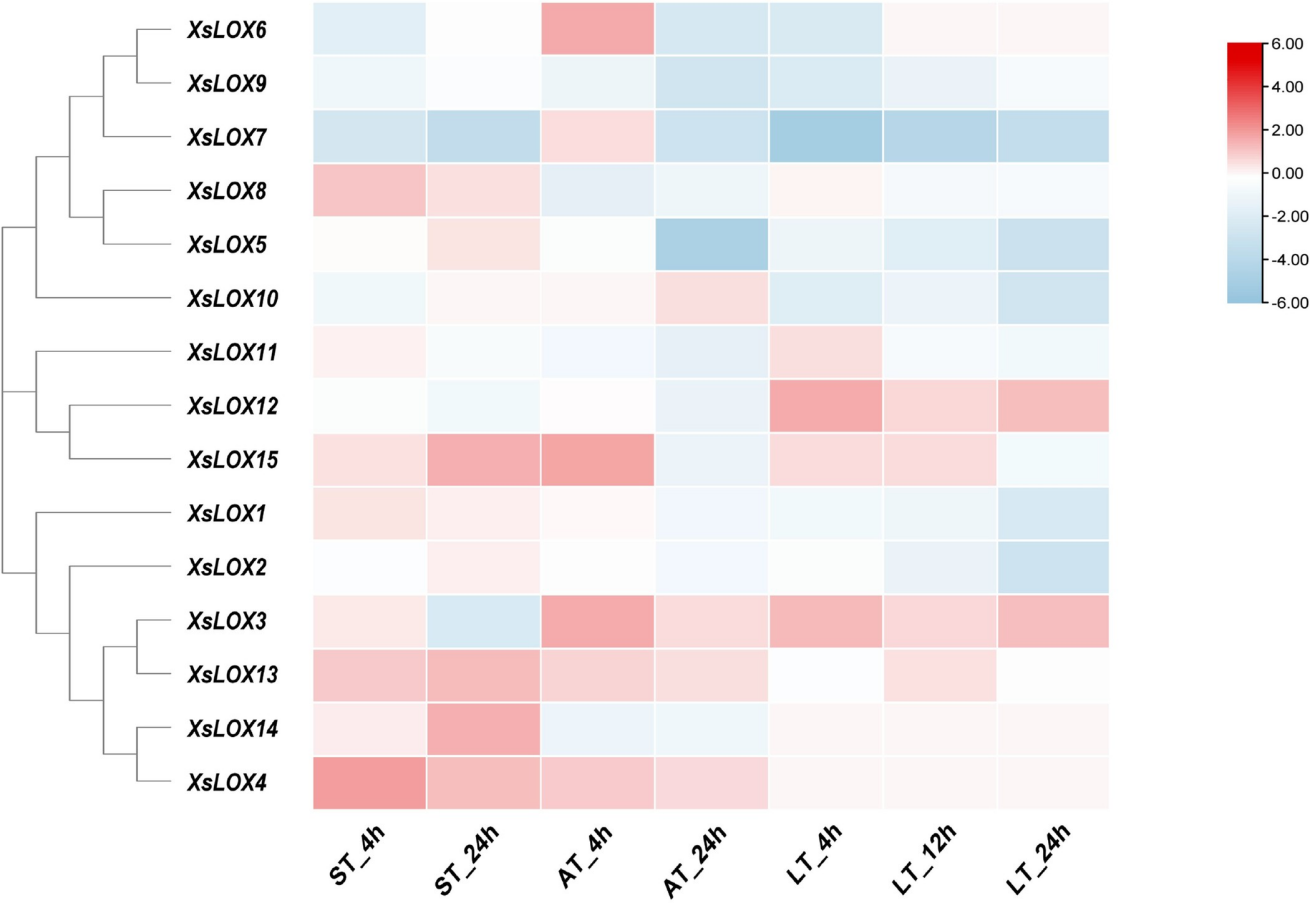
The differential expression levels of the *XsLOX* genes in response to low temperature treatment (LT), salt treatment (ST), and alkali treatment (AT). The seedlings were treated with NaCl (150 mM) and Na_2_CO_3_ (150 mM) for 4 h and 24 h, low temperature (4°C) for 4 h, 12 h, and 24h. Red squares indicate increased abundance and blue squares indicate decreased quantity.

The expression levels of each *XsLOX* gene were evaluated using RNA-seq data (**Fig 6**). When exposed to low-temperature stress, two *XsLOX* genes were upregulated, and five were downregulated at both 4 hours and 24 hours. Similarly, six *XsLOX* experienced decreased expressions, and four experienced increased expressions after 12 hours. By contrast, two and four genes were downregulated and upregulated when exposed to salt stress for 4 and 24 hours. Upon alkali stress for 4 hours, three *XsLOX* genes decreased, and three increased in expression. In addition, during the 24-hour stress-induced period, eight *XsLOX* remained downregulated, with none upregulated. Finally, one *XsLOX* gene was downregulated, and five upregulated after the application of drought. Among them, the three *XsLOX* genes (*XsLOX5/ 7/ 9*) co-expressed and downregulated in response to all treatments. These findings demonstrate that *XsLOXs* are pivotal actors in abiotic stress-related processes.

### qRT-PCR analysis of LOX

**Fig 7:**
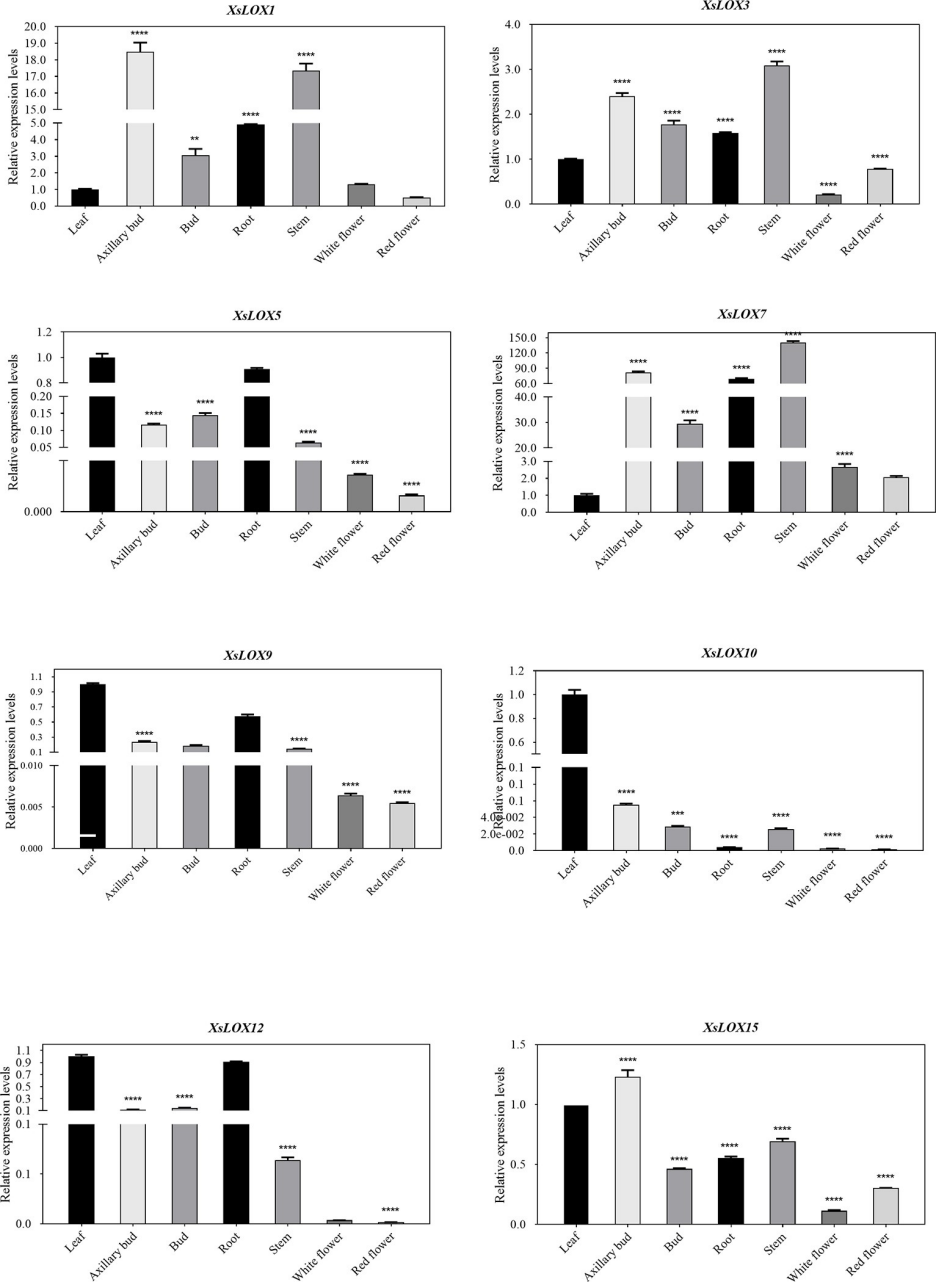
Changes in expression analysis of eight randomly chosen *XsLOX* genes in seven tissues of mature plants by qRT-PCR. All qRT-PCR data were normalized against that of the housekeeping gene. X-axis shows different tissues, and Y-axis shows the relative expression level. Error bars represent the mean ±SE of three biological replicates, and asterisks represent a significant difference from Duncan’s method (*p = 0.05, **p = 0.01, ***p = 0.001, ****p = 0.0001).

To further understand the expression pattern of the *XsLOX* gene in yellow horn, expression profiles of the *XsLOX* gene in seven different tissues were examined from qRT-PCR data (**Fig 7**). In standardizing relevant expression data, leaf expression is used as a reference. *XsLOX1*, *XsLOX3*, and *XsLOX7* were highly expressed in roots, stems, axillary buds, and buds, while relatively few existed in other tissues. In particular, *XsLOX7* was the highest expression level in flowers and stems, indicating that the *XsLOX* gene mainly plays a role in these tissues. It is worth noting that *XsLOX5*, *XsLOX9*, *XsLOX10*, *XsLOX12*, and *XsLOX15* showed opposite expression patterns, and only *XsLOX15* had slightly higher expression levels in axillary buds. The results of qRT-PCR showed that *XsLOX* had a significant tissue-specific expression, indicating the different roles in plant development.

**Fig 8:**
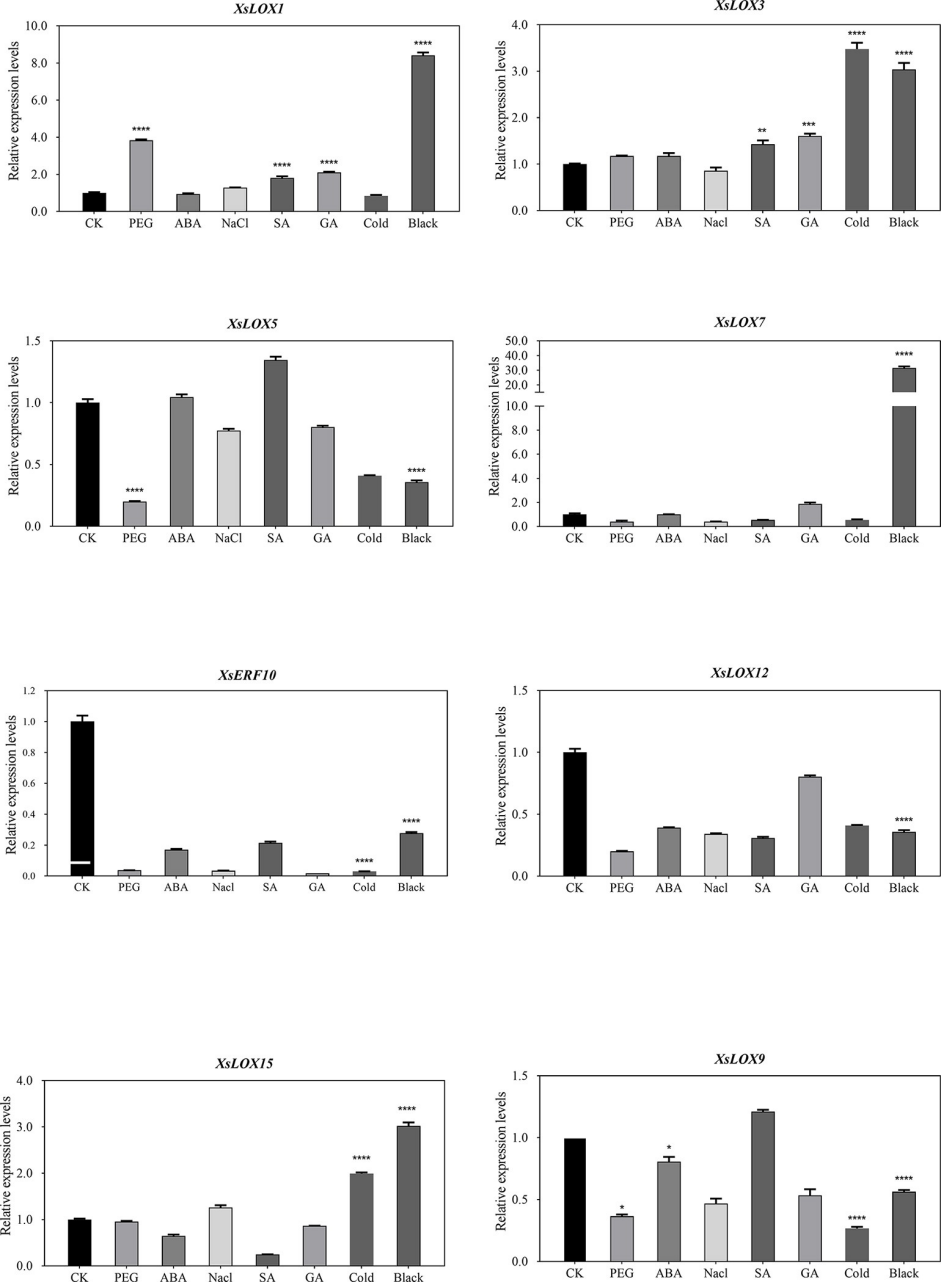
Changes in expression analysis of eight randomly chosen *XsLOX* genes in response to 10% PEG 6000, 100 mM NaCl, 4°C low temperature, 100 μM ABA, 200 μM SA, and 100 μM GA stress for 24 h and dark for 48 h. X-axis shows the different stress, and Y-axis shows the relative expression level. Error bars represent the mean ±SE of three biological replicates, and asterisks represent a significant difference from Duncan’s method (*p = 0.05, **p = 0.01, ***p = 0.001, ****p = 0.0001).

qRT-PCR was used to verify the expression pattern of *XsLOX* under abiotic stress and hormone. After drought treatment, *XsLOX1* was up-regulated, *XsLOX5* and *XsLOX9* were down-regulated, and other genes were not significantly expressed (**Fig 8**). The expression of *XsLOX5/9/10/12* (type II 13-LOX) was up-regulated considerably under dark and low-temperature treatment. *XsLOX7* is highly expressed under dark processing. After salt treatment, only *XsLOX3* was upregulated. The expression levels of *XsLOX1* and *XsLOX3* have been up-regulated after SA and GA treatment, but no significant induction of any *XsLOX* genes was observed after ABA treatment. *XsLOX* genes play a potential role in combating abiotic stress and responding to exogenous plant hormones.

## Discussion

Lipoxygenases have been the subject of intensive study in various organisms, playing a critical role in growth and development, signaling molecule generation, stress response, and plant defense [3, 17, 38, 39]. Through the utilization of HMM and BlastP combining the annotation data from yellow horn, fifteen potential *XsLOX* genes have been uncovered, surpassing those found in *Arabidopsis thaliana* [40] and tea plant [41], foxtail millet [16], tartary buck wheat [42], *Passiflora Edulis* [27], tomato [12], maize [43], but still be less than those discovered in *Salvia miltiorrhiza* bung [44], banana [45], apple [9], cucumber [39], cotton [15] and *Populus* [46]. An evolutionary analysis revealed that the four *XsLOX* proteins comprising *XsLOX2/3/4/13/14* assemble into the 9-LOX subfamily; *XsLOX1* falls under the 13-LOX I subfamily, with *XsLOX5/6/7/8/9/10/11/12/15* conforming to the 13-LOX II subfamily [5, 9]. The familial evolution of genes has been primarily ascribed to gene duplication through consecutive repetition events. Evolutionarily, variance in genetic phylogenetic trees may suggest conflict in active roles, while genes clustered on the same branch may have a similar function.

*XsLOX3/4* and *XsLOX5/6/7/8/9* are disposed of in tandem on chromosomes 3 and 7, respectively. The evolution of genes has been predominantly correlated to the duplication of the gene through consecutive replication events. This system may produce new genes and sub-activities, devising complementary non-redundant uses with the ancestral genes. Tandem echo can take place for multiple motives. It is assumed that unequal chromosomal crossings are the main catalysts of duplication events and the generation of genetic disparities among the species.

It is postulated that the presence of tandem repeated genes facilitates the organism’s adaptability to changing conditions, playing an essential part in evolutionary parsimony. Tandem repeats are believed to contribute to the prokaryote organism’s resilience and invulnerability, a claim corroborated by the fact that the expression of LOX genes is involved [47]. The Ka/Ks ratio was observed to be inferior to one, thus providing pointers for research related to the genes of yellow horn immunity [48].

Subcellular localization prediction analysis of *XsLOX* indicated that type LOX II proteins possess an N-terminal plastid transporter and are localized in the chloroplast, consistent with the related family clustering outcomes. In contrast, type LOX I proteins are mainly found in the cytoplasm, lacking the transporter [49].

The gene structure and lipoxygenase domain were highly conserved in *XsLOX* genes. To gain insight into how the gene is regulated under various environmental conditions, we analyzed promoter cis-acting elements of each *XsLOX* gene family. Our findings indicate cis-elements were classified into MeJA response elements, gibberellin response elements, low-temperature response elements, drought-inducibility elements, circadian control, light-responsive elements, and hormonal pathways. These outcomes align with those observed in other species [13], with the most light-sensitive features.

LOX plays an important role in the growth and development of yellow horn. The expression levels of some genes detected by transcriptome sequencing did not match the qRT-PCR analysis. By qRT-PCR analysis, it was found that the expression levels of one down-regulated gene *XsLOX*12 under low-temperature stress were different from the RNA-Seq data. LOX can catalyze both enzymatic and non-enzymatic pathways to synthesize oxidized lipids. The expression of this enzyme and its gene underlies the synthesis of these lipids and stress responses. It is speculated that this gene’s expression may result from its involvement in specific metabolic pathways correlated with the synthesis of certain molecules. The *AtLOX1* gene has been demonstrated to be critical in maintaining defensive responses in Arabidopsis leaves [22]. *AtLOX5* has been identified as highly expressed in the roots and appears to be involved in lateral root morphogenesis [14]. Studies have indicated that enhanced 9-LOX catalysis of 9S-HPOT synthesis, with its derivatives, has been linked to the dynamics of lateral root formation and could thus reduce primary root growth [22, 50]. Tomato *TomLoxB* and *TomLoxC* positively regulated ethylene content during fruit ripening [51, 52]. The application of jasmonic acid promoted the expression of *ZmLOX6* in maize [53]. JA and SA induce the expression of *Gllox1* and *Gllox2* in *Gracilariopsis lemaneiformis* [54]. In our study, SA induces the expression of *XsLOX1* and *XsLOX3*. In injured potatoes and rice, ABA can stimulate LOX activity [55, 56]. The *GhLOX* gene plays an important role in the development of the nutrient tissue of cotton [15]. Expressions of *XsLOX1*, *XsLOX3*, and *XsLOX7* were significantly elevated in axillary buds, stems, and roots, suggesting that these genes may have important functions in these tissues.

## Conclusions

In this study, 15 *XsLOX* genes were identified, exhibiting highly conserved gene structures and being divided into two subgroups and unevenly distributed across a single chromosome. Analysis of their differential and specific expression under heat, cold, drought, and salt stress and subsequent qPCR verification revealed they play a significant role in responding to abiotic stresses. Moreover, *XsLOX* genes were found to be particularly active in tissue specificity, which may be correlated to their structural similarity among family members and differences in the cis-acting elements. Consequently, our findings provide new insights into the crucial activities of the *XsLOX* gene across evolution, development, and abiotic stress tolerance.

## Supporting information

S1 Table q-PCR primers. (PDF)

S2 Table List of *XsLOX* genes identified. (PDF)

S3 Table Motif 1 contains the conserved sequence His-(X) 4 -HIS-(X) 4 -HIS-(X) 17 -HIS-(X) 8 -HIS. (PDF)

S4 Table Estimation of the Ka/Ks ratio of homologous gene pairs in yellow horn. (PDF)
