# Supplementary material for: Identification and characterization of lipoxygenase (LOX) genes involved in abiotic stresses in yellow horn
Source: PLoS One. 2023 Oct 13;18(10):e0292898. doi: 10.1371/journal.pone.0292898 (PMC10575502; doi:10.1371/journal.pone.0292898)
Supplement: S1 Table — (PDF) [file pone.0292898.s001.pdf]

Primer name	Sequence 5' to 3'	PCR product length
UBC2-FP	ATTGGAGATGGAACTGTA	122
UBC2-RP	TTCAACTGGTAGATACGA	
XsLOX1-FP	TCGATTGCAGGTCTTGGGTG	98
XsLOX1-RP	CGCTTTAGGTGTGTCGCTTG	
XsLOX3-FP	TTTGGGTGATCCCGACAAGG	191
XsLOX3-RP	TCAAGTGACCAAACCGCTCA	
XsLOX5-FP	TCACCGGAGTCAGCAATCAC	163
XsLOX5-RP	ATAGGGTTGTGCCTTCGAGC	
XsLOX7-FP	AGTATGTGGGCGATAAGGCG	155
XsLOX7-RP	CGTCCCAGCTCCATTTCTGT	
XsLOX9-FP	TCGGACACGGTGACAAGAAG	129
XsLOX9-RP	ACTGGCCAAAGTTCACTGCT	
XsLOX10-FP	TAGCAGGGGATTGGCTGTTG	158
XsLOX10-RP	CAAGGCTTGGGTCAGGGTAG	
XsLOX12-FP	GTCCGGCGACACAGAGATAA	102
XsLOX12-RP	TTCAGGTTCGGATCACCGTT	
XsLOX15-FP	AAAACGTGTGGACGGTGAGA	98
XsLOX15-RP	TGTTGTGGTGGTGTGTTTGTG	
