# Supplementary material for: Identification and characterization of lipoxygenase (LOX) genes involved in abiotic stresses in yellow horn
Source: PLoS One. 2023 Oct 13;18(10):e0292898. doi: 10.1371/journal.pone.0292898 (PMC10575502; doi:10.1371/journal.pone.0292898)
Supplement: S2 Table — (PDF) [file pone.0292898.s002.pdf]

Gene ID	Gene name	Chromosome position	CDS (bp)	GC _counts	PI	MW (KDa)	Length (aa)	Subcellular localization
EVM0017987.1	*XsLOX1*	X.sor_LG1	2,493	1,097	6.39	94,820.46	831	nucleus
EVM0006060.1	*XsLOX2*	X.sor_LG3-1	2,649	1,203	6.00	101,074.31	883	cytoplasm
EVM0013716.1	*XsLOX3*	X.sor_LG3-2	2,613	1,200	5.60	99,019.37	871	cytoplasm
EVM0009464.1	*XsLOX4*	X.sor_LG3-3	2,553	1,143	5.43	97,094.16	851	cytoplasm
EVM0015511.1	*XsLOX5*	X.sor_LG7-1	2,709	1,217	5.70	102,187.98	903	chloroplast
EVM0004614.1	*XsLOX6*	X.sor_LG7-2	2,721	1,211	6.29	103,359.71	907	chloroplast
EVM0015334.1	*XsLOX7*	X.sor_LG7-3	2,703	1,218	6.42	102,203.92	901	chloroplast
EVM0004686.1	*XsLOX8*	X.sor_LG7-4	2,709	1,224	5.72	102,202.03	903	chloroplast
EVM0009328.1	*XsLOX9*	X.sor_LG7-5	2,610	1,158	5.95	99,370.03	870	chloroplast
EVM0020815.1	*XsLOX10*	X.sor_LG7-6	2,721	1,239	6.34	102,627.99	907	chloroplast
EVM0002061.2	*XsLOX11*	X.sor_LG8	2,781	1,245	7.70	104,527.56	927	chloroplast
EVM0016818.1	*XsLOX12*	X.sor_LG9	2,760	1,297	6.69	103,672.3	920	cytoplasm
EVM0024359.1	*XsLOX13*	POS55864-1	2,613	1,200	5.57	98,969.31	871	cytoplasm
EVM0015141.1	*XsLOX14*	POS55864-2	2,553	1,139	5.33	97,039.03	851	cytoplasm
EVM0012264.1	*XsLOX15*	POS71333	2,760	1,298	6.68	103,561.17	920	cytoplasm
