# Supplementary material for: Identification and characterization of lipoxygenase (LOX) genes involved in abiotic stresses in yellow horn
Source: PLoS One. 2023 Oct 13;18(10):e0292898. doi: 10.1371/journal.pone.0292898 (PMC10575502; doi:10.1371/journal.pone.0292898)
Supplement: S3 Table — (PDF) [file pone.0292898.s003.pdf]

Gene name	Gene star	Gene end	p-value	Site
XsLOX1	481	530	1.10E-54	HQLISHWLHTHAVVEPFIIATRRQLSVMHPIHRLLDPHFKDTMHINALAR
XsLOX2	533	582	1.73E-57	HQLISHWLNTHAVIEPFVIATNRQLSVLHPIYKLLQPHFRDTMNINALAR
XsLOX3	526	575	5.09E-56	HQLISHWLNTHAAIEPFVIATNRQLSVLHPIHKLLLPHFRDTMNINAFAR
XsLOX4	506	555	2.29E-55	HHLISHWLHTHAVIEPFVVAANRQLSVLHPIYKLLHPHFRDTMFINAFAR
XsLOX5	555	604	5.61E-64	HQLVSHWLRTHCCTEPYIIATNRQLSAMHPIYRLLTPHFRYTMEINALAR
XsLOX6	559	608	5.61E-64	HQLVSHWLRTHCCTEPYIIATNRQLSAMHPIYRLLTPHFRYTMEINALAR
XsLOX7	553	602	1.33E-62	HQLVSHWLRTHCCAEPYIIATNRQLSAMHPIYRLLTPHFRYTMEINALAR
XsLOX8	555	604	5.61E-64	HQLVSHWLRTHCCTEPYIIATNRQLSAMHPIYRLLTPHFRYTMEINALAR
XsLOX9	522	571	5.61E-64	HQLVSHWLRTHCCTEPYIIATNRQLSAMHPIYRLLTPHFRYTMEINALAR
XsLOX10	559	608	3.53E-65	HQLVSHWLRTHCCTEPYIIATNRQLSVMHPIYRLLHPHFRYTMEINALAR
XsLOX11	579	628	2.45E-57	HQLVNHWLRTHACMEPYIIAAHRHLSSMHPIFMLLHPHMRYTLEINALAR
XsLOX12	574	623	1.22E-57	HQLSNHWLRTHACMEPFILAAHRQLSAMHPIFKLLDPHMRYTLEINAQAR
XsLOX13	526	575	5.09E-56	HQLISHWLNTHAAIEPFVIATNRQLSVLHPIHKLLLPHFRDTMNINAFAR
XsLOX14	506	555	2.29E-55	HHLISHWLHTHAVIEPFVVAANRQLSVLHPIYKLLHPHFRDTMFINAFAR
XsLOX15	574	623	1.22E-57	HQLSNHWLRTHACMEPFILAAHRQLSAMHPIFKLLDPHMRYTLEINAQAR
