# Supplementary material for: Identification and characterization of lipoxygenase (LOX) genes involved in abiotic stresses in yellow horn
Source: PLoS One. 2023 Oct 13;18(10):e0292898. doi: 10.1371/journal.pone.0292898 (PMC10575502; doi:10.1371/journal.pone.0292898)
Supplement: S4 Table — (PDF) [file pone.0292898.s004.pdf]

Duplicated gene pairs	Ka	Ks	Ka/Ks
XsLOX6-XsLOX7	0.0946622	0.27210484	0.3478887
XsLOX5-XsLOX6	0.12536766	0.33949414	0.3692778
XsLOX8-XsLOX9	0.1337005	0.33856125	0.3949079
XsLOX3-XsLOX4	0.09740119	0.40385099	0.241181
